# Motor skills, cognitive impairment, and quality of life in normal pressure hydrocephalus: early effects of shunt placement

**DOI:** 10.1007/s00701-022-05149-2

**Published:** 2022-02-25

**Authors:** Matthias Hülser, Hannah Spielmann, Joachim Oertel, Christoph Sippl

**Affiliations:** Department of Neurosurgery, Faculty of Medicine, Saarland University, HomburgSaar, Germany

**Keywords:** iNPH, Hydrocephalus, VP-shunt, Neuro-psychological

## Abstract

**Background:**

Traditionally, clinical findings of normal pressure hydrocephalus are mainly characterized by the Hakim triad. The aim of this study is to evaluate the performance of patients suffering from idiopathic normal pressure hydrocephalus (iNPH) in a more holistic manner regarding motor skills, cognitive impairment, and quality of life.

**Methods:**

In total, 30 individuals diagnosed with iNPH as well as a reference group with another 30 individuals were included. The iNPH patients and the reference group were age, educational, and morbidity matched. A standardized test battery for psychomotor skills, gait, neuropsychological abilities as well as questionnaires for quality of life was applied. The iNPH group was tested prior to surgery, at 6 weeks, and 3 months postoperatively. The reference group was tested once.

**Results:**

Patients showed a significant improved performance in various items of the test battery during the first 3 months postoperatively. This included neuropsychological evaluation, motor skills including gait and upper motor function as well as the quality of life of the patients. Compared to reference individuals, neuropsychological aspects and quality of life of iNPH patients improved in some parts nearly to normal values.

**Conclusion:**

Our findings underline that shunt surgery does not only improve the symptoms in iNPH patients but also ameliorates the quality of life to a great extent close to those of age and comorbidity matched reference individuals. This data enables an optimized counseling of iNPH patients regarding the expectable outcome after shunt surgery especially regarding cognitive performance, motor skills as well as life quality.

## Introduction

Idiopathic normal pressure hydrocephalus (iNPH) is a degenerative disease of the brain typically characterized by the so-called Hakim triad presenting with gait disturbance, dementia as well as incontinence [10]. In CT or MR imaging, it is associated with enlarged ventricles and narrow apical sulci [16]. Most commonly, a spinal-tap test is conducted with gait assessment to approve diagnosis [49]. Pathological patterns in brain pressure monitoring also indicate iNPH and may serve as an adjuvant diagnostic tool [1]. Different treatment options such as endoscopic third ventriculostomy are discussed, but to this day the gold standard is drainage of CSF via shunt surgery [20, 48]. Considering comorbidities, the prognosis of shunt implantation for iNPH is considered good, at least temporarily.[22]

Yet when evaluating this very outcome, clinicians mainly rely on the improvement of the Hakim triad, a tribute to its straightforward accessibility [18]. As swift as this approach may be in clinical practice, it does not give credit to the whole symptom complex iNPH patients display with cognitive and life quality impairment [36, 37].

In the recent years, manifold additional tests were proposed to characterize the daily life performance of patients suffering from iNPH in a more holistic manner [37]. This includes neuropsychological tests, tests of cognitive function, sophisticated assessment of motor skills, as well as questionnaires regarding the performance in daily life. Huge credit must be given to Hellström as well as other authors who showed that additional test parameters predict outcome after shunt placement more accurately than Hakim triad and spinal-tap test alone [12, 13]. Unfortunately, most of these studies focus on the temporal dynamics within a cohort of iNPH patients after shunt implantation without a reference group of non-iNPH individuals. Thus, although it is well known that iNPH patients do significantly benefit from surgery, the extent of recovery in relation to non-iNPH reference individuals remains inconclusive.

The goal of the present study was to evaluate the effect of shunting in iNPH patients with a standardized test battery widened for neuropsychological aspects and fine motor skills. Subsequently, the effect of the test results on the quality of life was evaluated. Additionally, the results were compared to a corresponding group of age and comorbidity matched reference individuals.

## Methods

### Patients

All patients who underwent shunt placement for iNPH between January 2020 and February 2021 were prospectively included in this trial. Patients displayed symptoms of the Hakim triad together with brain imaging finding of enlarged ventricles. Diagnosis of iNPH was confirmed via spinal-tap test. Therefore, gait was assessed with 10-m walking test and 360° rotation. Consecutively, 30–40 ml of CSF was drained via lumbar puncture. Gait testing was repeated identically 30 to 60 min thereafter. If spinal-tap test was still inconclusive, telemetric measurement of brain pressure was conducted to confirm iNPH.

Inclusion criteria were as follow: (1) informed consent of the patient to participate in the study, (2) diagnosis of iNPH, (3) shunt placement. Altogether, 30 consecutive patients meeting these criteria were included. Clinical follow-up is available until April 2021.

Another 30 individuals with a comparable age, gender distribution, BMI, grade of academics as well as comorbidity for matched pair analysis served as a reference group. Details of the cohort can be found in Table 1. The study was approved by the local ethics committee (No. 147/20).

**Table 1:** Patient baseline characteristics

	iNPH Group (*n* = 30)	Reference Group (*n* = 30)	
**Mean age ± SD, [range] in years**	76.9 ± 5.6 [63.2–87.6]	77.9 ± 5.6 [66.0–91.7]	*p* = 0.552
**Gender, no, (%)**			
Male	17, (56.6%)	17, (56.6%)	*p* = 1.0
Female	13, (43.3%)	13, (43.3%)	
**BMI ± SD, [range] in (kg/m**^**2**^**)**	27.4 ± 2.9 [21.6–34.0]	27.8 ± 4.5 [19.5–41.0]	*p* = 0.715
**Death by end of trial, no., (%)**	1, (3.3%)	0, (0%)	
**Academics**	8, (26.6%)	8, (26.6%)	*p* = 1.0
**Comorbidity**			
Hypertension	22, (73.3%)	22, (73.3%)	*p* = 1.0
Diabetes	6, (20.0%)	6, (20.0%)	*p* = 1.0
Cardiac disease	13, (43.3%)	13, (43.3%)	*p* = 1.0
Peripheral vascular disease	5, (16.6%)	4, (13.3%)	*p* = 0.718
Prostate hyperplasia	5, (16.6%)	3, (10.0%)	*p* = 0.432
Osteoporosis	4, (13.3%)	2, (6.6%)	*p* = 0.389

*SD* standard deviation.

### Surgical procedure

The patients were operated in the supine position under general anesthesia. Small incisions were made at the right Kocher’s point, behind the right ear and paraumbilically. The shunt system included a pump reservoir and two valves. The first valve in line was a differential pressure valve, opening at a magnetically adjustable, pressure value. The second valve in line contained an antisiphon device. The whole system was tunneled from the head to the abdomen. The ventricular catheter was placed in the lateral ventricle via a burr hole. Using the pump reservoir, the system was tested in situ. If the shunt worked properly, the abdominal catheter was placed intraperitoneally.

### Brain imaging

Prior to surgery as well as postoperatively, all patients underwent brain imaging including CT scan as well as MRI. Preoperative MRI secured the diagnosis of iNPH with enlarged ventricles and narrow apical sulci. Furthermore, an obstructed CSF flow was excluded as etiology of hydrocephalus via time-resolved 2D phase-contrast imaging with velocity encoding sequences. On the first postoperative day, a CT scan ensured the correct position of the intraventricular catheter. An X-ray confirmed the intraperitoneal position of the abdominal catheter. At 3 months postoperatively, a CT scan was conducted during follow-up.

### Testing battery

With the testing battery, the typical symptoms of the Hakim triad like gait ataxia, dementia, urinary incontinence as well as specific neuropsychological findings were assessed. The individual tests are described in the following paragraph.

To grade the severity of personal restrictions or disabilities concerning everyday life, the Modified Rankin Scale subdivided in six different grades was applied [40].

The Stein and Langfitt questionnaire was used to evaluate the clinical conditions regarding the aspect of coping with everyday life [44].

Evaluation of continence following the Hellström et al. score was rated in six grades [13]. Patients who are suffering from iNPH are usually elderly people, so the occurrence of comorbidities is high.

Kiefer et al. introduced a scale in which four different risk factors, vascular, cerebrovascular, cardiac, and others like Parkinson disease, were rated [22]. For additional information apart from the three typical symptoms, the Kiefer score added headache and dizziness. This score gives the opportunity to grade each symptom according to the respective severity. The final score is the sum of each point of the symptoms [21].

The EQ5D Test is subdivided in five different dimensions: mobility, self-care, usual activities, pain/discomfort, and anxiety/depression. Those dimensions are subdivided into three grades, in specific no, some, and extreme problems [3, 7].

To evaluate the general cognition and memory performance, the Mini Mental Status Examination (MMSE) and the DemTect were performed. [5, 8] The DemTect is a screening tool for dementia, testing intellectual flexibility, awareness, and verbal memory. It is especially sensitive for mild and beginning forms of dementia. [19]

Verbal episodic memory, learning, long-term recall, and word recognition were assessed with Rey Auditory Verbal Learning Test (RAVLT).

To measure executive frontal lobe-related functions such as flexibility, speed of processing, and conceptual abilities as well as impulse control, the Stroop Test with two different parts was included: Stroop Test A—the color naming test and Stroop Test B—the interference test. During Stroop A, the name of the four different colors squares needs to be reproduced as fast as possible. Stroop B is more difficult; not the name of the word, rather the written color of the word needs to be reproduced as fast as possible [45].

For evaluation of the short-term memory, the Digit Span test forward (A) and for the working memory, the Digit Span Test backward (B) were used. In part A, the examiner reads out a sequence of numbers; after every right enumeration, the sequence gets one number longer. In part B, the number must be recalled backwards in the right sequence.

To evaluate psychomotor speed, visual search and correct assignment, attention as well as mental flexibility, the Trail Making Test (TMT) was applied. Using TMT A, 25 numbers need to be connected in the right sequence. During TMT B, numbers and letters should be connected alternately, e.g., 1,A,2,B etc.[2]

For evaluation of complex coordinative demands as well as fine motor skills, the grooved pegboard test was assessed. This consists of 25 differently arranged holes, five per row. Beginning with the dominant hand, for example the right hand, the pegs need to be placed from left to right. Using the other hand, the pegs need to be placed in the other direction [13].

Gait ataxia was evaluated identical to spinal-tap test effect (see above). Steps were counted, and the time was measured in seconds.

Finally, the finger-tapping test counted the maximum finger taps possible in 10 s measuring the motor speed of the index finger on each hand [42].

### Statistical analysis

All statistical analyses were performed using SPSS v.25 (IBM, Armonk, USA). Results of the psychometric tests were coded as standardized z-scores according to the test norms of the respective manuals. *X*^2^, analysis of variance (ANOVA) as well as independent Student *t* test were used to compare the different groups. A value of *p* < 0.05 was considered statistically significant. A value of *p* < 0.10 was considered a statistical trend. Standard deviation is presented by ± . Range is presented in squared brackets [].

## Results

In the following paragraph, results of the testing are displayed. Results are grouped in three categories: neuropsychological testing, motor skill testing, and quality of life assessment. In the text, the best performance during follow-up is highlighted. At the end of this section, complications and radiological results are listed.

### Neuropsychology

**Fig. 1:**
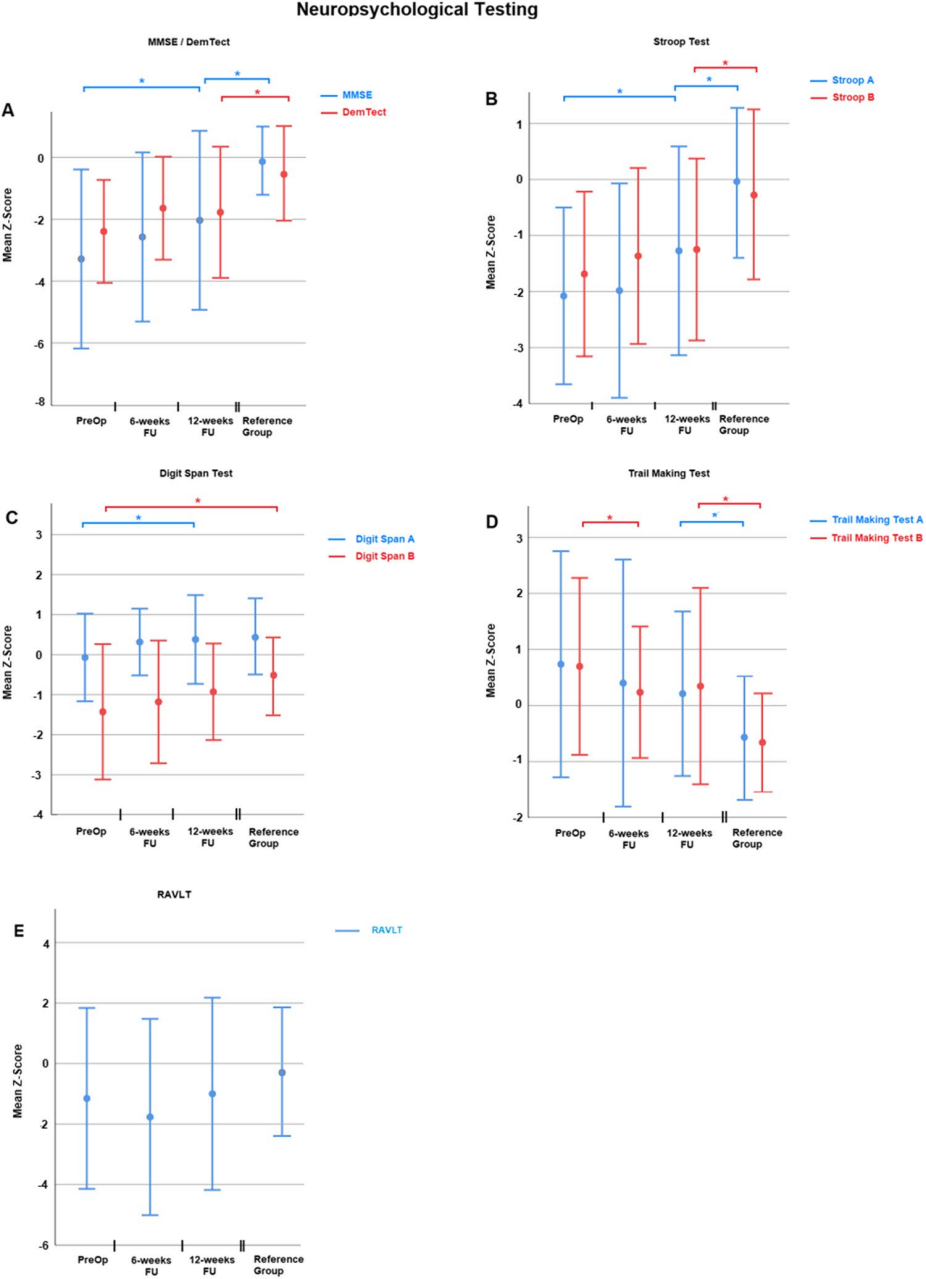
Summarizes the results of neuropsychological testing as z-scores. In all sections on X-axis, different times of testing are depicted. Y-axis represents the z-score. Section **A**, Results of MMSE (blue) and DemTect (red). Section **B**, Results of Stroop A (blue) and B (red). Section **C**, Results of Digit Span A (blue) and B (red). Section **D**, Results of Trail Making Test A (blue) and B (red). Section **E**, Results of RAVLT (red). The bars represent the standard deviation

The MMSE, the DemTect, the Stroop A and B, the Trail Making Test A and B as well as the RAVLT assessed neuropsychological performance. Details of the testing can be found in Table 2 and Fig. 1. It must be noted that a lower z-score in Trail Making Test A and B represents a better clinical performance. In contrast is a better performance of all other neuropsychological tests associated with a higher z-score. In MMSE, Stroop A, Trail Making Test B, and Digit Span A, the performance of patients improved significantly during follow-up. The results of the DemTect also improved on a trend level 12 weeks after surgery compared to preoperatively (*p* = 0.06). Regarding MMSE, DemTect, Stroop A and B as well as Trail Making Test, A and B individuals of the reference group performed nevertheless significantly better than iNPH patients at their best follow-up. After 12 weeks post-surgery, no difference between reference group and iNPH patients could be detected in Digit Span A and B and RAVLT.

**Table 2:** Results of neuropsychological testing

	**z-score (**mean ± SD)				***p* value**	
Test	Pre-op	6-week FU	12-week FU	Reference group	Pre-op vs 12-week FU	12-week FU vs reference group
MMSE	− 3.3 ± 2.8	− 2.5 ± 2.7	− 2.0 ± 2.8	− 0.1 ± 1.1	0.024	0.001
DemTect	− 2.4 ± 1.6	− 1.6 ± 1.6	− 1.8 ± 1.8	− 0.5 ± 1.4	0.15	0.012
Digit Span A	− 0.1 ± 1.0	0.3 ± 0.8	0.4 ± 1.1	0.4 ± 0.9	0.024	0.85
Digit Span B	− 1.4 ± 1.6	− 1.1 ± 1.5	− 0.9 ± 1.2	− 0.5 ± 0.9	0.095	0.16
Stroop A	− 2.1 ± 1.5	− 1.9 ± 1.9	− 1.2 ± 1.8	− 0.1 ± 1.3	< 0.001	0.006
Stroop B	− 1.7 ± 1.4	− 1.4 ± 1.5	− 1.2 ± 1.6	− 0.3 ± 1.5	0.062	0.021
TMT A	0.7 ± 2.0	0.4 ± 2.2	0.2 ± 1.4	− 0.5 ± 1.0	0.17	0.027
TMT B	0.7 ± 1.5	0.2 ± 1.1	0.3 ± 1.7	− 0.6 ± 0.8	0.059	0.009
RAVLT	− 1.5 ± 3.1	− 1.8 ± 3.2	− 1.2 ± 3.1	− 0.3 ± 2.1	0.6	0.2

### Motor skills

**Fig. 2:**
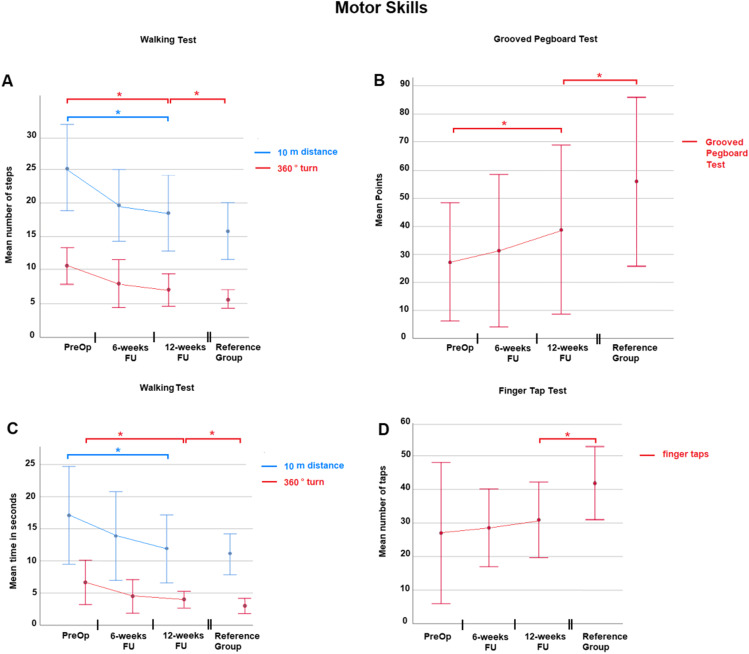
Visualizes the results of testing of motor skills. In all sections on X-axis, different times of testing are depicted. Section **A**, Number of steps needed in walking test. Ten-meter walking distance is highlighted in blue, 360° turn in red. Section **B** highlights results of the grooved pegboard test. Time used is depicted on Y-axis. Section **C**, Time in seconds needed in walking test. Ten-meter walking distance is highlighted in blue, 360° turn in red. Section **D** visualizes the finger-tapping test. On Y-axis number of taps achieved is depicted

Motor skills were assessed with 10-m walking distance and 360° turn analysis as well as grooved pegboard test and finger-tapping test. Details can be found in Table 3 and Fig. 2. Comparing preoperative performance with the 12-week follow-up, iNPH patients improved significantly in the walking test and grooved pegboard test. The walking time and steps needed for a 10-m distance were not significantly different between iNPH patients and the controls 12 weeks after surgery. The other tests of motor skills revealed that despite improvement iNPH patients performed significantly worse than individuals of the reference group.

**Table 3:** Results of motor skills testing

						*p* value	
Test	Unit	Pre-op	6-week FU	12-week FU	Reference group	Pre-op vs 12-week FU	12-week FU vs reference group
**Walking test**							
10 distance	steps ± SD	25.0 ± 6.4	19.6 ± 5.3	18.5 ± 5.6	15.7 ± 4.2	0.001	0.059
360° turn		10.5 ± 2.7	8.1 ± 3.5	7.0 ± 2.4	5.6 ± 1.3	0.001	0.02
10 distance	s ± SD	17.1 ± 7.5	13.8 ± 6.8	11.9 ± 5.1	10.8 ± 2.9	0.009	0.363
360° turn		6.6 ± 3.4	4.6 ± 2.7	3.9 ± 1.3	3.1 ± 1.1	0.004	0.01
**Grooved pegboard test**	points ± SD	27.0 ± 21.0	31.3 ± 27.3	38.3 ± 30.1	56.0 ± 30.1	0.002	0.03
**Finger-tap test**	taps ± SD	26.5 ± 13.2	28.6 ± 11.6	31.0 ± 11.1	42.0 ± 10.3	0.06	0.001

### Quality of life

**Fig. 3:**
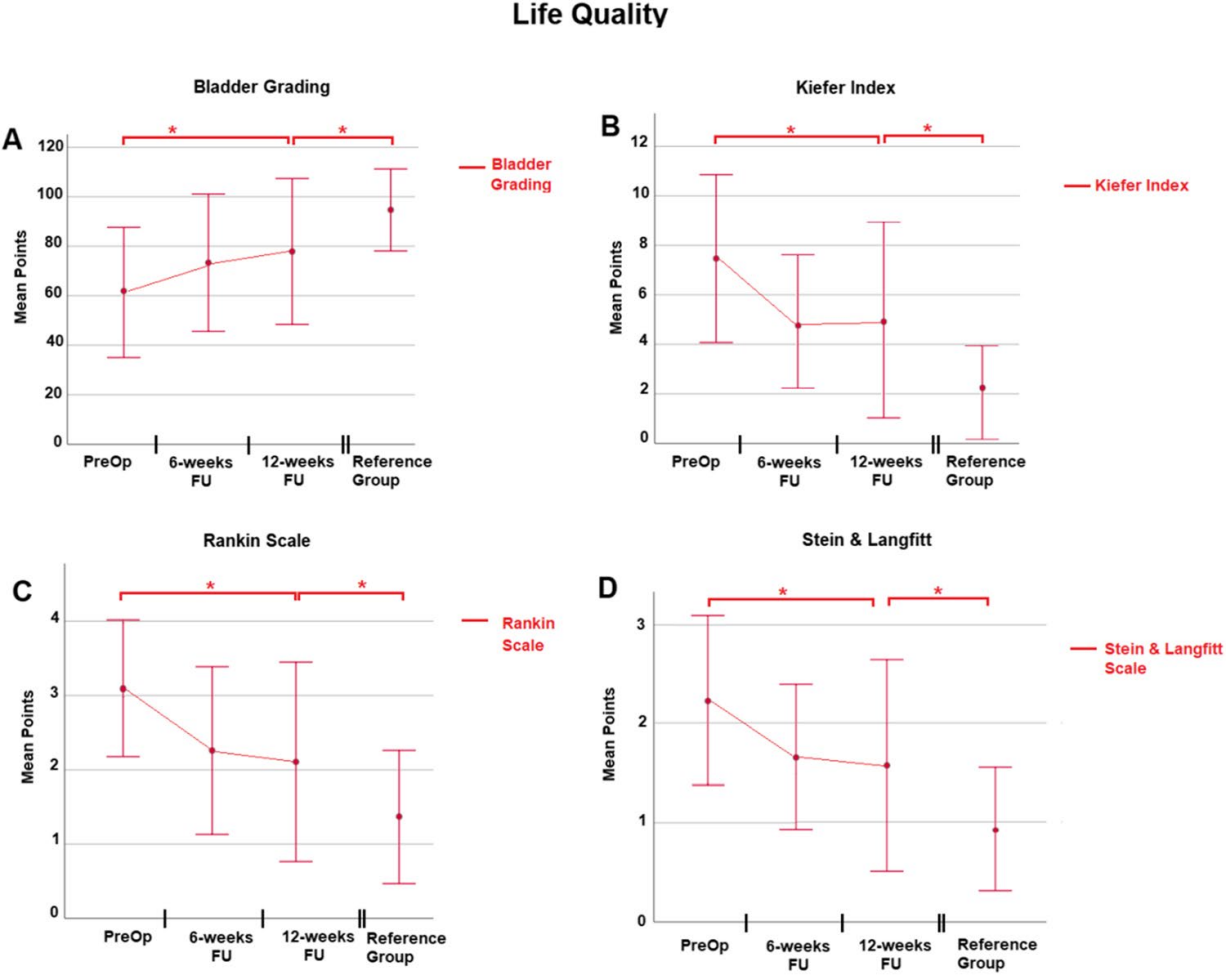
Highlights quality of life assessment. In all sections on X-axis, different times of testing are depicted. Section **A**, Bladder grading. Y-axis represents the mean points achieved in each test. Section **B**, Kiefer Index. Y-axis represents points in Kiefer grading. A high number is associated with impaired performance. Section **C**, Results of Rankin Scale. Y-axis represents points gathered. A high number is associated with impaired performance. Section **D**, Results of Stein and Langfitt assessment. Y-axis represents points gathered. A high number is again associated with impaired performance

**Fig. 4:**
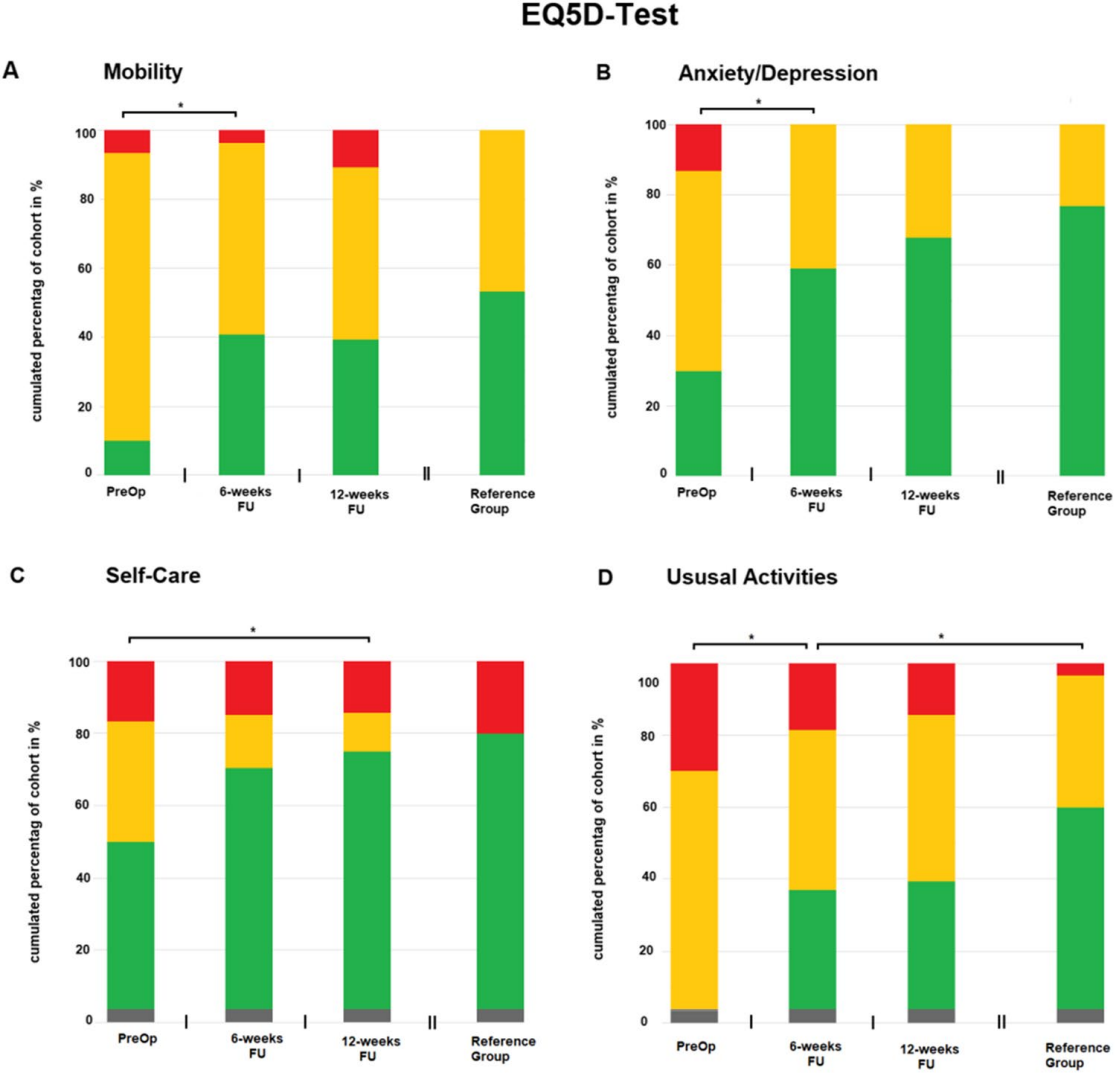
Depicts results of EQ5D assessment. In all sections, X-axis represents different times of testing. Y-axis represents the cumulated percentage of the cohort in percent. Color green represents Level 1 in EQ5D, good performance. Orange represents Level 2, medium performance. Red represents Level 3, bad performance. Section **A**, **B**, **C**, and **D** highlight Mobility, Anxiety/Depression, Self-Care, and Usual activities, respectively

The quality of life was assessed in manifold aspects encompassing bladder function, Kiefer Index, Rankin Scale, and Stein and Langfitt Scale as well as EQ5D Test. Details can be found in Table 4 and Figs. 3 and 4. Bladder function, Kiefer Index, Rankin Scale, and Stein and Langfitt Scale improved significantly at the 12-week follow-up. In all these aforementioned tests, individuals of the reference group performed significantly better. Regarding EQ5D Test, a significant improvement in iNPH patients after shunting regarding mobility (*p* = 0.003), self-care (*p* = 0.043), usual activities (*p* = 0.001), pain/discomfort (*p* = 0.001), and anxiety (*p* = 0.001) could be found. In mobility, self-care as well as anxiety, patients reached performance not statistically distinguishable from individuals of the reference group. Regarding usual activities as well as pain, the reference group performed significantly better compared to iNPH patients even at their best follow-up.

**Table 4:** Results of quality-of-life assessment

	Mean points ± SD				*p* value	
Test	Pre-op	6-week FU	12-week FU	Reference group	Pre-op vs 12-week FU	12-week FU vs reference group
**Bladder grading**	61.3 ± 26.2	73.3 ± 27.7	77.9 ± 28.9	94.7 ± 16.5	0.015	0.009
**Kiefer Index**	7.5 ± 3.4	4.9 ± 2.7	4.9 ± 3.8	2.0 ± 1.9	0.001	0.001
**Rankin Scale**	3.1 ± 0.9	2.2 ± 1.1	2.1 ± 1.3	1.4 ± 0.9	0.001	0.016
**Stein and Langfitt**	2.2 ± 0.9	1.6 ± 0.7	1.6 ± 1.1	0.9 ± 0.6	0.001	0.006

### Complications

In this cohort, three patients needed another surgery, indicating a complication rate of 10%. One patient suffered a dislocation of the distal catheter 11 months after shunting, needing revision surgery. Two patients developed subdural hygromas because of over-drainage 3 and 5 months after shunting. In both patients, the subdural hygroma was evacuated via a burr hole.

## Discussion

### Summary of results

Patients showed a significant improved performance in various items of the test battery during the first 3 months postoperatively. This included neuropsychological evaluation, motor skills including gait and upper motor function as well as the quality of life of the patients. Compared to reference individuals, neuropsychological aspects and quality of life of iNPH patients improved in some parts nearly to normal values.

### Neuropsychology

In theory, impairment of cognition in patient with iNPH results from increased degradation of paraventricular and subcortical structures with consecutive dysfunction of subcortical pathways and tracts. Especially in the earlier stages of the disease, a wide range of neurophysiological deficits occurs which corresponds to a subcortical-frontal dementia. These deficits affect psychomotor speed, attention, concentration, working memory, spatial and constructive ability, flexibility of thinking, and executive functions [6, 28, 31, 46]. But also verbal learning and memory, which are temporal lobe functions and typically deteriorated in Alzheimer’s disease, are decreased in patient with iNPH [12, 34].

When it comes to shunt treatment, cognitive deficits are considered to be the least likely to improve compared to gait disturbance or even to urinary incontinence [9, 29]. However, a 2016 published meta-analysis of the effect of shunt surgery on neuropsychological function found evidence for improvement in some cognitive domains, such as global cognitive function, verbal learning, memory, and psychomotor speed [37]. Despite this evidence, detailed data on the improvement of executive function remain inconsistent until this date*.*[37] Certainly, there are a few well-designed studies evaluating executive function testing after shunt treatment; however, they only focus on a specific domain of the widespread facets of the cerebral executive functioning [12, 28].

Due to the wide range of executive function, a set of four different tests was used for assessment. The (backwards) Digit Span Test B evaluates the working memory as the essential part of all executive functions [27]. The Trail Making Test B examines flexibility and productivity of thinking. The Swedish Stroop Test A and B are used for the assessment of naming speed and response selection as well as inhibition respectively and represent therefore a complex verbal learning test [12, 45].

We found that the working memory of the iNPH patients after shunt surgery did not improve significantly 3 months after shunting. However, the significant difference of the working memory found preoperatively in iNPH patients compared to that of reference subjects decreased after shunt surgery. Also, flexibility and productivity of thinking did improve in these patients but did not reach the level of the reference group. The same observation applies for the executive functions of naming speed as well as response selection and inhibition. One possible explanation is that there is an irreversible impairment of frontal function in normal pressure hydrocephalus.

The Mini Mental State Examination (MMSE) and the DemTect are both screening tests for diagnosing dementia and include an evaluation for different memory domains as well as other cognitive areas [8, 19].

The MMSE assesses, besides memory, attention, and orientation, other basic cognitive domains such as reading, writing, calculating, visuo-spatial functioning, speech production, and speech comprehension [8]. In this study, the patients improved significantly 3 months after surgery but did not reach the baseline of the reference individuals. This result matches those reported in literature where an overall improvement of performance in MMSE is described [37]. Yet, there are also studies that did not find a benefit in cognition tested by MMSE [14, 39]. Especially in patients performing in or near the “normal range” above 24 points, there were no significant changes found [14]. This could be explained with a “ceiling effect” which is due to a reduced sensitivity of MMSE for mild cognitive impairment [38].

Therefore, being the more sensitive tool in detecting mild cognitive impairment, the DemTect was added to the test battery of this study [19]. Generally, studies about the effectiveness of cognitive assessment using the DemTect in patient with iNPH are rare.

The DemTect evaluates short-term, working and semantic memory as well as attention and verbal fluency. Also, executive functions like cognitive flexibility, problem solving, speed of processing, language production, and language comprehension are evaluated [19]. Interestingly, the patients did not significantly improve in the DemTect after surgery in the first 3 months. Therefore, the usefulness of the DemTect in the assessment of cognitive function after shunt surgery remains unclear.

Regarding verbal learning, verbal long-term memory, and short-term memory, our results are disappointing. In literature, the Rey Auditory Verbal Learning Test (RAVLT) seems to be sensitive for memory improvement [37]. Additionally, the test is useful for the prediction of cognitive enhancement of shunt therapy, if there had been prior improvement after spinal-tap testing [30]. Even though Hellström et al. described the RAVLT as feasible in 90% of his iNPH patient cohort [12], in our cohort it turned out to be rather poorly feasible for the majority of patients. Therefore, the delayed recall section of the test was removed from our standardized test battery. One explanation could be that the cognitive impairment of our patient cohort might have been greater than that of the study by Hellström et al. The reasons for this discrepancy will remain unclear since there was no cognitive assessment performed by Hellström et al.

### Motor skills

iNPH is typically a frontal disorder characterized by wide-based, slow, and magnetic gait [41]. However, it can also include a wide spectrum of different gait phenotypes from normal or unspecific to parkinson-like[4, 32] and the different gait profiles seem to respond to a different extent to shunt therapy [33]. Nevertheless, generally the gait disorder is the symptom of the Hakim triad which responds best to shunt therapy [9, 29].

Expectedly, the present cohort of iNPH patients showed a significant improvement of gait ataxia, assessed by a 10-m walk and a 360° turn. This applies for both the amount of steps and the time needed. Three months after surgery, the 10-m walk test of the iNPH patients did not differ from the walk of the reference group. From a practical point of view, testing the amount of steps from the 360° turn alone is a sufficient and easy applicable test for monitoring the effect of shunt therapy.

Nevertheless, gait testing might not be feasible for patients with an advanced gait disturbance. In these cases, testing the upper motor function is an alternative.

Even though the traditional view is that the motor dysfunction in patient with NPH can be summarized in a lower-body Parkinsonism, a few studies showed that psychomotor speed and dexterity of the upper limbs are also affected [11, 26, 43, 47].

Based on our results, the grooved pegboard test was a suitable test for monitoring the effect of shunt treatment on the upper limb motor disturbance. The feasibility of performing it is quite high, but there is the need to purchase the test materials. Therefore, the finger-tapping test was introduced to our test battery. This test helps to distinguish between healthy patients and patients with motor dysfunctions of cerebellar, basal ganglia, and cerebral origin [42].

To our knowledge, there is only one other study using this test in patient with iNPH, monitoring the effect of shunt treatment on psychomotor speed of the upper limbs. In this study, the authors could demonstrate a significant improvement in the finger-tapping test 1 month after shunt surgery [26]. In our study, a trend toward an enhanced motor ability was seen 3 months postoperatively (*p* = 0.06); however, it did not reach a statistically significant threshold. Further studies are needed to quantify the usefulness in patient with iNPH.

### Quality of life

The overall outcome and disability of everyday life of such patients (Rankin Scale and Stein and Langfitt Scale) as well as specific symptoms of the disease (Kiefer Index) improve due to shunt therapy 3 months after surgery but do not reach the level of the reference group. In this cohort, bladder function improved significantly at 3 months postoperatively. Even though there was an improvement, the reference group performed significantly better concerning bladder function. Urinary incontinence in patient with iNPH is with up to 91% a common symptom [23]. At least one part of the urinary dysfunction seems to be because of detrusor overactivity [23]. Shunt treatment leads to a significant improvement in urinary urgency and urge incontinence which also affects positively the overall quality of life [24].

Though, shunt surgery leads to an amelioration of the whole iNPH symptom complex, the gain of quality of life has to be assessed with different tools. Therefore, the EQ5D Test was chosen which is recommended for patients with mild to moderate cognitive deficits [35]. The patients benefitted from shunt therapy regarding mobility, self-care, usual activities, pain/discomfort, and anxiety. Furthermore, in the domain mobility, self-care as well as anxiety, the patients even reached a level which is comparable to the individuals of the reference group. The results of the three previous studies evaluating QoL in patient with iNPH are inconclusive since there are different statements regarding the extent of amelioration [17, 25, 36]. However, a more recent published study demonstrated that the increase in QoL can endure also over a long period of time (at least 21 months) post-surgery [15]. Although the preoperative QoL was raised retrospectively, the study included a large cohort of patient (176 patients) with a representative reference group. In their evaluation, the patient cohort did not reach the level of QoL of the reference group. In contrast the present study, demonstrated no difference in QoL at 3 months after surgery.

### Limitations of the study

The authors are aware that the low number of patients in the iNPH group limits the generalization of the results. Furthermore, are only early results after shunt placement reported, the follow-up is limited to 12 weeks postoperatively. Hence, it is not sure if the amelioration of the performance of the iNPH patients remains stable in the continuing course of the disease.

## Conclusion

In this prospective study, an overall improvement in all the fields assessed could be shown after shunt surgery. Not only motor and cognitive functioning improved, but also the patients’ quality of life improved significantly and consecutively after surgery according to their own perception. Parts of the QoL were similar to those of an age and education matched reference group at 3-month follow-up. These findings underline that shunt surgery does not only improve the symptoms in iNPH patients but also ameliorates the quality of life to a great extent close to those of reference individuals. The next step would be a long-term study to harden the findings at hand.

## Data Availability

The used tested battery and analyzed data during the current study are available from the corresponding author on reasonable request.

## Abbreviations

**BMI**: Body mass index
**iNPH**: Normal pressure hydrocephalus
**CSF**: Cerebrospinal fluid
**CT**: Computed tomography
**DemTect**: Dementia detection
**MMSE**: Mini Mental State Examination
**MR**: Magnetic resonance imaging
**QoL**: Quality of life
**RAVLT**: Rey Auditory Verbal Learning Test
**TMT**: Trail Making Test
